# Risk factors for immune-related adverse events: what have we learned and what lies ahead?

**DOI:** 10.1186/s40364-021-00314-8

**Published:** 2021-11-03

**Authors:** Xiaoyan Liu, Yuequan Shi, Dongming Zhang, Qing Zhou, Jia Liu, Minjiang Chen, Yan Xu, Jing Zhao, Wei Zhong, Mengzhao Wang

**Affiliations:** 1 Department of Respiratory and Critical Care Medicine, Peking Union Medical College Hospital, Chinese Academy of Medical Sciences & Peking Union Medical College, No.1 Shuaifuyuan Wangfujing, Dongcheng District, 100730 Beijing, China; 2 Department of Respiratory and Critical Care Medicine, Peking Union Medical College Hospital, Chinese Academy of Medical Sciences & Peking Union Medical College, No 1 Shuaifuyuan Wangfujing, Dongcheng District, 100730 Beijing, China

**Keywords:** Immune checkpoint inhibitor, Immune-related adverse events, Mechanism, Risk factor

## Abstract

Immune checkpoint inhibitors (ICIs) have heralded the advent of a new era in oncology by holding the promise of prolonged survival in severe and otherwise treatment-refractory advanced cancers. However, the remarkable antitumor efficacy of these agents is overshadowed by their potential for inducing autoimmune toxic effects, collectively termed immune-related adverse events (irAEs). These autoimmune adverse effects are often difficult to predict, possibly permanent, and occasionally fatal. Hence, the identification of risk factors for irAEs is urgently needed to allow for prompt therapeutic intervention. This review discusses the potential mechanisms through which irAEs arise and summarizes the existing evidence regarding risk factors associated with the occurrence of irAEs. In particular, we examined available data regarding the effect of a series of clinicopathological and demographic factors on the risk of irAEs.

## Introduction

Cancer immunotherapy has shifted the treatment paradigm for a variety of malignant tumors, including melanoma, lung cancer, bladder cancer, head and neck cancer, lymphoma, kidney cancer, Merkel cell tumors and microsatellite instability-high cancer [1]. The most thoroughly studied immunotherapy uses immune checkpoint inhibitors (ICIs), which include anti-programmed cell death receptor-1 (PD-1)/programmed death ligand-1 (PD-L1) and anti-cytotoxic T-lymphocyte–associated antigen 4 (CTLA-4) treatment. CTLA-4 and PD-1 are immune checkpoints that negatively regulate T-cell immune function.

Although checkpoint blockade immunotherapy can enable the host immune system to recognize and destroy tumor cells, cross-reactivity with self-peptides may cause side effects. Therefore, the release of these natural brakes may affect the self-tolerance to healthy tissues, producing immune side effects known as immune-related adverse events (irAEs). These toxicities vary in severity and across organ systems, manifesting as a variety of conditions, such as rash, pruritus, vitiligo, encephalitis, hypophysitis, uveitis, thyroiditis, pneumonia, myocarditis, hepatitis, pancreatitis, colitis, nephritis, and musculoskeletal and hematologic disorders [1]. The frequency of irAEs varies with the agents used and the susceptibility of individual patients.

With the increasing use of ICIs, the spectrum of irAEs has expanded beyond common presentations involving dermatological, gastrointestinal and endocrine systems to rarer manifestations, such as nervous and hematopoietic toxicities, which constitute a challenge for the diagnosis and monitoring of irAEs in clinical settings. Adding to the challenge is that some irAEs, if not detected early, can cause irreversible or even fatal damage. Therefore, exploring the mechanisms underlying irAEs and identifying predictive biomarkers for irAEs are essential to maximize anticancer benefit while minimizing irAE risk. This article discusses our current immunologic understanding of irAEs and examines the available evidence regarding risk factors associated with the development of irAEs.

## Safety profiles of ICIs

The incidence of any-grade irAEs in patients receiving the anti-CTLA-4 antibody ipilimumab is up to 65 %, with diarrhea and/or colitis being the most common (occurring in approximately 20 % of patients) [2, 3]. Grade ≥ 3 irAEs occur in approximately 5–30 % of patients and commonly emerge within 8–12 weeks after treatment initiation, with skin rash usually having the earliest onset [3]. Less frequent irAEs (occurring in less than 20 % of patients) include hepatitis, pruritus and endocrinopathies, such as hypophysitis and thyroiditis. Other rare toxicities (arising in < 2 % of patients) include episcleritis, uveitis, pancreatitis, nephritis, sarcoidosis-like reactions, autoimmune thrombocytopenia, toxic epidermal necrolysis and Stevens–Johnson-like syndromes and neuropathies, such as myasthenia gravis, autoimmune autonomic ganglionopathy, and Guillain–Barré syndrome (GBS) [2, 4–7]. The occurrence of irAEs is temporally correlated. Early onset irAEs usually affect the skin or mucosa, such as colitis, rash and pneumonitis, which primarily occur after the second cycle of immune therapy [3, 8]. In contrast, irAEs that affect specific organs, including hypophysitis, vitiligo, and hepatitis, are reported as late onset adverse events (AEs) [9, 10].

Existing data suggest that the risk of irAEs in patients receiving ipilimumab is dose- and duration-dependent [4, 6]. In a phase III trial comparing the efficacy of a 10 mg/kg dose of ipilimumab with that of a 3 mg/kg dose in patients with advanced-stage melanoma, patients in the low-dose ipilimumab group had a decreased risk for grade ≥ 3 AEs (18 % versus 37 %) [4]. In another study where patients with high-risk stage III melanoma received 10 mg/kg doses of adjuvant ipilimumab on a 3-weekly and then 3-monthly basis for up to 3 years, 54 % of patients had grade ≥ 3 treatment-related AEs, suggesting that long-term treatment with a high dose of ipilimumab confers a high risk of irAEs [6].

In comparison with anti-CTLA-4 antibodies, irAEs caused by PD-1/PD-L1 inhibitors are less frequent and differ in severity and their spectrum of organ involvement. The incidence of any-grade irAEs and grade ≥ 3 irAEs is 13–40 % and approximately 10 %, respectively [3, 11–15]. The most frequently reported irAEs include endocrinopathies (mostly thyroiditis), pneumonitis, hepatitis, diarrhea and colitis [3, 11–15]. Existing data suggest that most irAEs related to anti-PD-1/PD-L1 antibodies emerge within the first 6 months of treatment [3]. Combined treatment with anti-CTLA-4 antibody and anti-PD-1 antibody increases both the incidence and severity of irAEs. The most likely affected organ is the colon (in 17 % of patients), followed by the skin, liver and endocrine system [11–15].

## Mechanisms underlying irAEs

The first step toward identifying irAE biomarkers is to understand the mechanisms that underlie the autoimmune effects. The pathophysiology of irAE remains poorly understood, although preclinical, translational, and clinical studies have provided some insight into potential mechanisms [16–51]. Hopefully, a better understanding of the immune process will facilitate the discovery of biomarkers for irAEs. However, it should be noted that much of what is known about the underlying mechanisms is derived from studies of autoimmune and autoinflammatory disorders.

First, blockade of immune checkpoints, which function to suppress self-reactive T cells, can disrupt central and peripheral tolerance. Both PD-1 and CTLA-4 are negative regulators of T cell activation, though the locations and timing of these regulatory events are different. Normally, CTLA-4 inhibits T cells at the initial stage of naïve T-cell activation in the early stages of the immune cycle in the lymph nodes [16, 17], while PD-1 regulates previously activated T cells at the later stages of an immune response in the peripheral tissues or at the tumor site [17]. The binding of CTLA-4 on naïve T cells to B7 on antigen presenting cells (APCs) generates an inhibitory signal during the primary phase of T cell activation [18]. In addition, CTLA-4 can induce transendocytosis of B7 molecules on APCs, which also inhibits T cell activation [19]. Furthermore, CTLA-4 is expressed on regulatory T cells (Tregs) and mediates T cell suppression [20]. In contrast, PD-1 interacts with its ligands PD-L1 and PD-L2 to deliver inhibitory signals later in the immune response in peripheral tissues [21]. In addition, PD-1 can also regulate signaling thresholds during T cell development and thus regulate central tolerance [22]. The distinct roles of PD-1 and CTLA-4 in maintaining immunological homeostasis are reflected in the different toxicity profiles observed in knockout mouse models. CTLA-4 gene knockout commonly results in severe lymphoproliferative disorders [23], whereas PD-1 gene knockout causes more limited and organ-specific toxicity [24]. The distinct mechanisms between anti-CTLA-4 antibody and anti-PD-1/PD-L1 antibody in developing irAEs are briefly summarized in Table 1.

**Table 1:** Different mechanisms of irAEs between anti-CTLA-4 antibody vs. anti-PD-1 antibody. Anti-CTLA-4 antibody generates inhibitory signal at early phase of T cell activation within lymph node, while anti-PD-1 antibody works mainly on peripheral tissue, at a comparatively late phase of immune reaction

	Anti-CTLA−4 antibody	Anti-PD−1 antibody
Functional time	Early	Late
Functional site	Lymph node	Peripheral tissue
Expression cell	Naive T cell, Treg	Effector T cell, Treg, B cell

In addition to boosting T-cell–mediated immunity, treatment with PD-1/PD-L1 inhibitors can also affect humoral immunity, either directly or indirectly. Initial studies indicated an immunosuppressive role of B cell subsets in murine models of autoimmune disease [25, 26]. Emerging data indicate that the PD-1^hi^ regulatory B-cell subset can suppress the tumor-specific T-cell response via IL10-dependent pathways upon PD-1/PD-L1 interaction [27]. Furthermore, Okazaki et al. found that PD-1 signaling inhibited BCR signaling by recruiting SHP-2 to its phosphotyrosine and dephosphorylating key signal transducers of BCR signaling [28]. Consistently, Thibult and colleagues demonstrated that blockade of PD-1/PD-L1 pathways increased B-cell activation, proliferation and secretion of immunoglobulin [29]. A study by Das et al. showed that combined treatment with anti-CTLA-4 and anti-PD-1 agents induced a significant decrease in the number of circulating B cells and an increase in plasmablasts. The combination therapy also resulted in an increase in the cluster of differentiation (CD) 21^lo^ B cell subset with a greater clonality as well as increased IFN-gamma signaling. In addition, an increase in plasma chemokine (C-X-C motif) ligand (CXCL) 13, a marker of germinal center activation in humans that is expressed on mature B cells, was observed in patients treated with combination therapy [30]. Furthermore, these changes in B cells correlated with both the frequency and timing of irAEs. Additionally, ICI treatment affects the production of autoantibodies. Indeed, mice genetically depleted of PD-1 developed lupus-like proliferative arthritis and glomerulonephritis with predominant IgG3 deposition [24]. De Moel and colleagues screened pretreatment and posttreatment samples of advanced melanoma patients treated with ipilimumab [31]. Among patients who were antibody negative before treatment, 19 % developed new autoantibodies, with anti-TPO (thyroperoxidase) and anti-TG (thyroglobulin) being the most common. In addition to inducing the production of new autoantibodies, ICI treatment may also facilitate pre-existing autoantibody-mediated autoimmunity. A study by Yukihiro and colleagues demonstrated that the presence of preexisting antibodies was independently associated with irAEs [32]. Osorio et al. also reported that autoreactive antibodies were associated with ICI-induced hypothyroidism and hypophysitis [33].

Another mechanism might be cross-presentation of shared antigens. As cytotoxic T-cells recognize tumor neoantigens and destroy tumor cells, the released antigen can be taken up and processed by APCs, which can in turn prime CD8 T-cells. This process is referred to as cross-presentation and is essential in the host antitumor immune response. However, in this process, nontransformed bystander cells can also be targeted. The death of bystander cells releases antigens that could be ingested by APCs and presented to T cells, thereby stimulating a second wave of T cells that can attack normal tissue. Two cases of fulminant myocarditis caused by combined treatment with ipilimumab and nivolumab have been reported, which lends support to the abovementioned hypothesis [34]. Tumors in both patients expressed muscle-specific antigens, and similar T-cell clones were found in both the myocardium and the tumor in one patient [34]. Similar to this finding, vitiligo, an autoimmune pigmentary disorder caused by an autoimmune attack on melanocytes, is frequently observed in melanoma patients receiving ICI treatment [35]. In another study of NSCLC patients treated with anti-PD-1 antibodies, TCR clonotype analysis was performed on matched biopsy samples of skin and tumor from 4 patients who developed dermatologic IRAEs [36]. Shared T-cell clones between skin and tumor were observed, and further in vitro study showed that the shared antigens between skin and tumor elicit T-cell responses in stimulated peripheral blood mononuclear cells from patients with dermatologic irAEs [36].

In addition, epitope spreading (ES) caused by immunotherapy-induced inflammation may also participate in the onset of irAEs. ES, is defined as the diversification of epitope specificity from the initial dominant epitope-specific immune response by original effector T cells both intramolecularly and intermolelcularly [37]. By diversifying epitope specificity, ES enhances the antitumor response thus leading to the recognition of self-antigens and loss of tolerance. It has been reported that ES occurs in patients receiving tumor vaccines [38], adoptive cell transfer therapy [39] or anti-CTLA-4 treatment [40].

The same is observed in renal irAE, which is mediated by the direct binding of the antibody to the PD-L1 expressed on renal tubular epithelial cells.

**Fig. 1:**
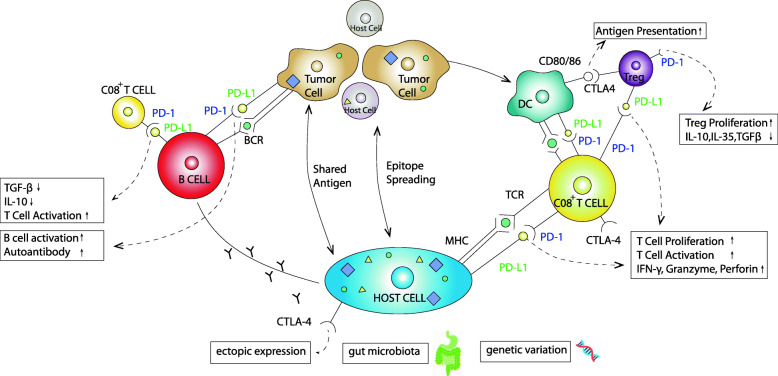
The mechanisms for immune-related adverse events (irAEs) remain elusive. Proposed mechanisms include enhanced activity of immune cells (primarily T and B cells) and consequent increased production of cytokines and antibodies against antigens that are shared between tumors and healthy tissue, as well as the ectopic expression of cytotoxic T-lymphocyte antigen 4 (CTLA-4) on normal tissue. Besides, the contribution of gut microbiota and genetic variability to irAEs suggest some other, as yet unspecified, mechanisms. Symbols including triangle, cycle and square in host cell and tumor cell represent antigens that each cell expressed

It is clear that not every patient receiving ICIs develops irAEs, and for those who do, their phenotypes and severity differ, suggesting that genetic profile and environmental exposure may be relevant as well. Given the role of genetics and genomics in autoimmune diseases [41], it is plausible that individuals genetically predisposed to autoimmunity may be more prone to develop irAEs. In addition, multiple lines of evidence indicate that the gut microbiota is intimately associated with the response to ICI treatment and the development of irAEs [42–45]. Finally, organ-specific expression of immune checkpoints may in part contribute to the development of organ-specific irAEs. For example, the commonly reported endocrine irAE following anti–CTLA-4 therapy with ipilimumab is hypophysitis, a side effect that is rarely observed after PD-1-antibody therapy, which may be partially due to complement-mediated inflammation via direct binding of an antibody with CTLA-4 expressed on pituitary cells [46, 47]. We created a diagram of irAE development, including the mechanisms mentioned above, which is shown in Fig. 1. The same is observed in the renal irAE, which is mediated by the direct binding of the antibody to the PD-L1 expressed on renal tubular epithelial cells [48–50].

Although irAEs are thought to be primarily T cell mediated, irAEs involve different organs and emerge at different times during treatment and may therefore have different primary mechanisms. Early onset irAEs are more like an autoinflammatory response that is caused by systemic immune dysregulation, such as acneiform or follicular ‘rash’. In contrast, late onset irAEs may be more of an autoimmune reaction. The recognition of shared antigens by tumor-specific T cells has been proposed as an underlying mechanism of delayed irAE by breaking down organ-specific tolerance [51].

Last, it should be noted that toxicity caused by ICIs is distinct from that caused by cytotoxic agents. In most cases, irAEs are not caused by the direct damage inflicted upon self-tissues; rather, they result from the enhanced immunity caused by ICIs and the shared antigen between tumor and host tissue, the pre-existing antibody, or the exposure to viral infection and concurrent medications during treatment with ICIs.

## Risk factors for irAEs

Compared to predictors for response, potential predictors for irAE occurrence have been less thoroughly investigated. Currently, we do not have clinically validated biomarkers to predict irAEs. However, significant strides have been made in identifying risk factors for irAEs. We have outlined potential risk factors along with the supporting evidence for each of them below.

**Table 2:** Risk factors for irAE. irAE, immune-related adverse event; IL-6, interleukin-6; NSCLC, non-small cell lung cancer; RCC, renal cell carcinoma; ILD, interstitial lung disease; GI, gastrointestinal; RLC, relative lymphocyte count; RNC, relative neutrophil count; REC, relative eosinophil count; CEACAM1, carcinoembryonic antigen cell adhesion molecule 1; AEC, absolute eosinophil count; DM, diabetes mellitus

Risk factor	Cancer type	Treatment	irAE	Ref
Body Composition Parameters				
Low muscle attenuation	Melanoma	CTLA-4 inhibitor	Grade III-IV irAE include rash, diarrhoea, colitis, hypotituitarism, arthritis etc.	[52]
Sex				
Female	Melanoma	CTLA-4 inhibitor	Grade III-IV irAE include pruritus, diarrhea, hypophysitis etc.	[53]
Tumour histology				
Melanoma compared to NSCLC		CTLA-4 inhibitor & PD-(L)1 inhibitor	↑Risk of GI & skin irAE↓Risk of penumonitis	[54]
Melanoma compared to RCC		CTLA-4 inhibitor & PD-(L)1 inhibitor	↑Risk of dermatitis, arthritis & myalgia↓Risk of pneumonitis & dyspnea	
RCC compared to melanoma		CTLA-4 inhibitor & PD-(L)1 inhibitor	↑Risk of pneumonitis & dyspnea	
Past medical history				
pre-existing AID	Solid tumors	CTLA-4 inhibitors & PD-(L)1 inhibitors	Likely increase risk of irAE, particularly in patients on immuno-suppressive therapy	[55–62]
HIV infection	Solid tumors	CTLA-4 inhibitors & PD-(L)1 inhibitors	Not increased	[63–71]
Concurrent or sequential treatment				
High dose radiotherapy	Solid tumors	CTLA-4 inhibitors & PD-(L)1 inhibitors	Likely increase risk of irAE, particularly in patients receiving higher radiation dose	[72–76]
Vemurafenib	Melanoma	CTLA-4 inhibitors	Hepatic toxicity	[77]
Trametinib + Vemurafenib	Melanoma	CTLA-4 inhibitors	Severe colitis	[78]
BRAF & MEK inhibitor	Melanoma	PD-(L)1 inhibitors	Hepatic toxicityPyrexia	[79, 80]
Crizotinib	NSCLC	PD-(L)1 inhibitors	Hepatic toxicity	[81, 82]
EGFR-TKI except osimertinib	NSCLC	PD-(L)1 inhibitors	Not increased	[83, 84]
Osimertinib prior to ICIs	NSCLC	PD-(L)1 inhibitors	Grade 3–4 irAEs include pneumonitis, hepatitis, colitis	[85]
Osimertinib after ICIs	NSCLC	PD-(L)1 inhibitors	Increased rate of ILD	[85–87]
Antibodies				
Pretreatment anti-thyroid antibody	NSCLC	PD-(L)1 inhibitors	Thyroid dysfunction	[32, 33]
Pre-treatment rheumatoid factor			Skin reactionAmyasthenia	[32]
Cytokine assays				
↑baseline IL-17	Melanoma	CTLA-4 inhibitors	Grade 3 colitis	[88]
↓IL-10 after treatment	Urothelial carcinoma	CTLA-4 inhibitors	ischemic papillopathy & optic neuritis	[89]
↓IL-10 and↑IL-2 after treatment	Melanoma	CTLA-4 inhibitors	irAE	[90]
↑baseline IL-6(P<0.05)	Melanoma	CTLA-4 inhibitors	Grade III-IV irAE include rash, diarrhoea, colitis, hypotituitarism, arthritis etc.	[53]
↓Baseline IL-6, IL-8, &sCD25	Melanoma	CTLA-4 inhibitors	Colitis	[91]
↑IL-6 after treatment	Melanoma	PD-(L)1 inhibitor	Cutaneous irAE	[92]
↓Baseline CXCL9, CXCL10, CXCL11 and CXCL19;↑Post-treatment CXCL9 and CXCL10;	Solid tumors	PD-(L)1 inhibitor & CTLA-4 inhibitor	Pneumonitis, thyroid, arthritis, dematitis, etc.	[93]
↑Circulating sCD163	Melanoma	PD-(L)1 inhibitor	irAE	[94]
↑Baseline sCTLA-4 level	Melanoma	CTLA-4 inhibitor	Gastrointestinal irAE	[95]
Blood cells				
↑WBC↓RLC↑RNC	Melanoma	PD-(L)1 inhibitor	GI irAEPulmonary irAE	[96]
↑CD177 & CEACAM1 (neutrophil-activation markers)	Melanoma	CTLA-4 inhibitor	GI irAE	[97]
ALC > 2000;↑Baseline AEC	Solid tumors	PD-(L)1 inhibitor	irAE include rash, colitis, hepatitis, pneumonitis, etc.	[98]
↑baseline AEC at and REC after treatment	Melanoma	PD-(L)1 inhibitor	Endocrine irAEs	[99]
Circulating T cells repertoire	prostate cancer	CTLA-4 inhibitor	IrAEs	[100]
Clonal expansion of CD8 T cells clones in peripheral blood	prostate cancer	CTLA-4 inhibitor	IrAEs?	[101]
Gut Microbiome				
Faecalibaterium and other firmicutes	Melanoma	CTLA-4 inhibitor	Colitis	[91]
B.fragilis and Burkholderia cepacia	murine sarcoma	CTLA-4 inhibitor	↓ Intestinal irAE	[43]
Bacteroidetes phylum	Melanoma	CTLA4 inhibitor	↓ colitis	[45]
Genetic Variability				
Predominance of HLA-DR4	Solid-organ cancer	PD-(L)1 inhibitor	diabetes	[102]
Homozygous variant of PDCD1 804 C>T(rs2227981)	NSCLC	PD-(L)1 inhibitor	↓ irAEs	[103]

### Clinicopathological and demographic characteristics

#### Body Composition Parameters

Body composition refers to the proportional content of body fat mass and lean body mass that can lead to a continuum of different phenotypes ranging from cachectic/sarcopenic state to obesity. Previous studies have demonstrated that sarcopenia is associated with poor survival and a higher incidence of adverse events in cancer patients who underwent chemotherapy or targeted therapy [104–106]. Consistent with early findings, recent studies found that sarcopenia was associated with worse treatment outcomes in non-small-cell lung cancer patients receiving anti-PD-1/PD-L1 therapy [107, 108]. Daly and colleagues investigated the association between body composition and irAEs in melanoma patients treated with ipilimumab. Multivariate analysis revealed that low muscle attenuation was significantly associated with high-grade irAEs [52]. Although the precise mechanism is unknown, several studies have suggested that sarcopenia may be related to systemic inflammation, which may contribute to the development of irAEs [109–111]. Improved response rates and survival has been noted in patients with obesity across different tumor types who are treated with ICI including PD-1/PD-L1 inhibitors [112, 113]. However, lack of association between obesity and irAEs incidence has been found.

#### Sex

Immune system function is known to vary between sexes as a result of hormonal and genetic effects [114]. Women are more susceptible to a variety of autoimmune diseases (AIDs), including systemic lupus erythematosus (SLE), multiple sclerosis (MS), primary biliary cirrhosis, rheumatoid arthritis and Hashimoto’s thyroiditis [115]. The increased susceptibility to autoimmune disease in females is proposed to be caused by the lack of protective effects of testicular hormones, fluctuating levels of ovarian hormones, and sex chromosome effects [116]. With respect to immunotherapy, existing data show that males may benefit more from ICI treatment [117], whereas females were reported to be associated with higher rates of irAEs [53]. Although this phenomenon still lacks well-grounded mechanistic explanations, sex-specific factors involved in the onset of AIDs may play a role in the development of irAEs.

#### Tumor Histology

A recent meta-analysis that included 48 trials with 6938 patients receiving either anti-CTLA-4, PD-1, PD-L1 treatments or combination treatments found that tumor histology was associated with the irAE profile [54]. Compared to non-small cell lung cancer patients, melanoma patients had a higher frequency of skin and gastrointestinal irAEs but a lower frequency of pneumonia [54]. Regarding the comparison between patients with melanoma and those with renal cell carcinoma, dermatitis, arthritis and myalgia were more prevalent in melanoma patients, whereas pneumonitis and dyspnea were less common in melanoma patients [54]. Although the precise mechanistic role of histology remains elusive, these findings could be partially explained by the different tumor microenvironments and neoantigens in different cancer types.

#### Underlying Comorbidities

Because of concerns about enhanced toxicity and compromised efficacy, patients with preexisting AIDs have been largely excluded from clinical trials of immunotherapy. The lack of information on the efficacy and, more importantly, safety profiles of immunotherapy in such patients has led to a clinical conundrum. Although no prospective clinical trial has assessed treatment with ICIs in cancer patients with AIDs, anecdotal evidence suggests that immunotherapy is effective and tolerable in this group of patients [55–62]. The incidence of irAEs or exacerbation of a preexisting AID is reported to range from approximately 30 to 75 %, with mixed evidence on the association between immunosuppressive therapy, either before or during ICI therapy, and the risk of irAEs [55–62]. Most de novo irAEs or flare-ups of preexisting AIDs are manageable with discontinuation of ICI treatment and initiation of corticosteroids and immunosuppressants.

Similarly, nearly all clinical trials of ICIs excluded people living with HIV (PLWH) due to various concerns, including interactions of ICIs with antiretroviral agents, unknown implications for the T-cell repertoire, and potential opportunistic infections. A paucity of retrospective cohort studies and sporadic case reports have shown that ICIs can be safe and effective in PLWH with cancer [63–71]. Although available data have described an acceptable safety profile for the use of ICIs in PLWH, concerns should be raised with regard to potential enhanced immune toxicity due to reversed immunity induced by treatment with ICIs [118–120], which is similar to the immune reconstitution inflammatory syndrome (IRIS), an overly robust antigen-specific inflammatory reaction observed in HIV patients undergoing dysregulated immune reconstitution after initiation of antiretroviral therapy [121].

Growing evidence indicates a role of viral infection in reshaping the tumor immune microenvironment [122–126]. Available data from clinical studies have shown that human papillomavirus (HPV) infection is associated with higher response rates and prolonged survival in patients with head and neck cancer treated with PD-1/PD-L1 inhibitors [127, 128]. Theoretically, the altered host immune response may also affect the development of irAEs. However, there are no published data so far on the association between viral infection and the risk of irAEs.

#### Treatment Modality

Accumulating evidence suggests that the effects of chemotherapy, targeted therapy or radiotherapy synergize with those of ICIs. However, the enhanced antitumor effect of combined therapy may come at the cost of augmented treatment toxicities.

Radiotherapy can induce immunogenic cell death and enhance the priming and activation of naïve T cells [129, 130]. However, at the same time, it can produce immunogenic damage to nonmalignant host cells and diversify the T cell receptor (TCR) repertoire, through which it will cause irAEs. Data from a handful of retrospective studies demonstrated that the addition of radiotherapy increased the incidence of irAEs, especially when radiation was given at higher doses [72–76]. While two of these studies showed that irAEs occurred outside of the radiation field and were attributed to ICI treatment alone[72, 73], the remaining studies suggested an association between the anatomical location of the irradiated site and immune toxicities [74–76]. However, one question that remains to be specified is whether organ-specific toxicities, especially pneumonia or pneumonitis in the setting of radiotherapy with concurrent or sequential immunotherapy are caused by radiation and then exacerbated by immunotherapy or initiated by immune damage from immunotherapy. In contrast, several studies denied the association of a heightened incidence of irAEs with radiotherapy [131–133]. Therefore, further studies need to be conducted to clarify whether the addition of radiotherapy to ICIs increases the risk of irAEs.

Data thus far have shown that some tyrosine kinase inhibitors (TKIs) may add to the toxicity of ICIs. The first study of this combination was a phase I study of the BRAF inhibitor vemurafenib with ipilimumab, which was stopped early due to severe hepatic toxicity [77]. In contrast, the combination of ipilimumab and another BRAF inhibitor, dabrafenib, was well tolerated. However, the addition of trametinib to this combination caused two cases of severe colitis leading to perforation in patients with metastatic melanoma. The combination of dabrafenib and trametinib has rarely been associated with colitis, and these two cases are presumed to be attributed to ipilimumab [78]. Additionally, anti-PD-1/PD-L1 monoclonal antibodies were assessed in combination with BRAF and/or MEK inhibitors [79, 80, 134, 135]. The toxicity of triplet therapy was higher than that known for either alone [79, 80].

Several clinical trials have explored combined therapy using PD-1/PD-L1 inhibitors and ALK or EGFR inhibitors in metastatic NSCLC. Treatment with PD-1/PD-L1 inhibitors plus crizotinib was investigated in patients with ALK-positive metastatic NSCLC [81, 82]. The results indicated enhanced liver toxicity with the combined treatment and do not warrant further investigation [81, 82].

The use of erlotinib plus atezolizumab or nivolumab did not demonstrate excess toxicity [83, 84]. However, an observational study from Oshima et al. raised the concern that the combination of nivolumab and EGFR-TKIs may be associated with a heightened risk of interstitial pneumonitis (IP) [136]. Furthermore, among the 18 patients with IP receiving both EGFR-TKIs and nivolumab, the sequence of administration was identified in 15 cases: all were treated with nivolumab followed by EGFR-TKIs. However, the specific EGFR-TKI used in those patients was not reported. Findings from a recent study provide deeper insight into the mechanisms of pulmonary toxicities [85]. Schoenfeld and colleagues found that six of 41 patients treated with sequential PD-1/PD-L1 inhibitor followed by subsequent osimertinib developed severe irAEs (four cases of grade 3 pneumonitis, one case of grade 3 colitis, and one case of grade 4 hepatitis), whereas no severe irAEs were observed in 29 patients receiving osimertinib followed by PD-1/PD-L1 blockade [85]. Consistent with previous studies [83, 84], no grade 3 or 4 toxicities were identified in 27 patients with sequential PD-1/PD-L1 inhibition followed by erlotinib. Severe irAEs were most common among those who initiated osimertinib within 3 months of prior anti-PD-1/PD-L1 therapy and required steroids and even biological agents. Kotake et al. also showed that interstitial lung disease (ILD) was seen in 21 % (4/19) of patients with the EGFR-T790M mutation-positive advanced NSCLC who received osimertinib shortly after prior nivolumab therapy [86]. Similarly, a phase Ib clinical trial of concurrent durvalumab plus osimertinib in EGFR-mutant lung cancer reported ILD in 38 % (13/34) of patients, bringing an early halt to the enrollment of patients in this arm [87]. Taken together, these findings suggest two things. First, the synergistic toxicity appears to be drug-specific rather than class-specific interactions of osimertinib with the PD-1/PD-L1 inhibitor. However, the exact mechanism of the interaction remains unclear. Second, the sequence and timing of therapy is an important determinant of the risk for irAEs. The impact of treatment sequence on irAEs may be due to the slow elimination and prolonged receptor occupancy of the anti-PD-1/PD-L1 antibody [137] and, in contrast, the relatively short half-life of osimertinib [138]. Hence, the use of osimertinib after anti-PD-1/PD-L1 antibody treatment heightens the likelihood of drug interaction.

With the increasing breadth of treatment options for advanced cancer patients, awareness of the risk of combined use of ICIs and other treatment approaches is needed to minimize toxicity and to optimally select and sequence available therapies.

#### Concurrent medications

As discussed in the previous paragraph, drug exposure has been shown to participate in the development of irAEs. Several studies have demonstrated association of the concurrent use of proton pump inhibitors, nonsteroidal anti-inflammatory drugs (NSAIDs) and a few other drugs with the development of ICI-induced acute interstitial nephritis (AIN) [139–142]. The potential mechanism could be that low-molecular weight drug compounds bind tubular antigens and thereby form a hapten trapped in the parenchyma that can elicit an immune response and cause tubular damage [143]. As such, exposure to drugs that are well tolerated prior to ICI therapy can initiate a drug-specific T cell-mediated immune response enhanced by immunotherapy. Similarly, it has been reported that patients who received antibiotics had a higher rate of ICI-induced diarrhea and/or colitis than those who received antibiotic therapy before ICI treatment [144].

### Preexisting Autoantibodies

Similar to T cells, PD-1 is also expressed in B cells and is involved in B cell activation, proliferation and the production of inflammatory cytokines [29, 145, 146]. Earlier studies in murine models revealed that PD-1 deficiency induces antibody-dependent immune toxicity [24, 147]. This suggests that the presence of autoantibodies may participate in the development of irAEs. Osorio et al. showed that anti-thyroid antibodies were present in 80 % of patients treated with pembrolizumab who developed thyroid dysfunction compared to 8 % of patients who did not [33]. Similar findings were reported in a recent study of NSCLC patients treated with nivolumab, where pretreatment antibodies and rheumatoid factors were more common among patients who developed irAEs. Multivariate analysis indicated that the presence of pretreatment antibodies was independently associated with irAEs. Furthermore, skin reactions were more frequent in patients with pretreatment rheumatoid factor, whereas thyroid dysfunction was more frequent in patients with pretreatment antithyroid antibodies [32]. Suzuki and colleagues examined 12 cases (0.12 %) of myasthenia gravis among 9869 cancer patients receiving nivolumab. Ten of 12 patients exhibited preexisting antibodies to the acetylcholine receptor [148].

### Cytokine Assays

Cytokines are essential for host immune function, affecting both innate and adaptive immune responses through involvement in immune cell proliferation, survival, differentiation, and regulation of effector functions [149]. A growing body of evidence suggests that cytokines also play important roles in orchestrating local immune homeostasis by promoting the recruitment of immune cells into the tumor microenvironment and inducing expression of immune checkpoint receptors [150–153]. Therefore, augmented expression of inflammatory cytokines may be linked to smoldering inflammation, which can be fueled by immunotherapy and develop into irAEs.

IL-17 (interleukin-17) is a proinflammatory cytokine that contributes to the pathogenesis of several autoimmune diseases, such as rheumatoid arthritis, multiple sclerosis, psoriasis, fibrotic lung diseases and inflammatory bowel disease [154–157]. IL-17 is secreted by Th17 cells, which abundantly express the coinhibitory molecule CTLA-4 [158]. Disruption of the IL-17 pathway may be involved in the development of irAEs. Tarhini et al. reported that elevated baseline IL17 levels were correlated with an increased incidence of grade 3 colitis in patients with melanoma treated with neoadjuvant ipilimumab [88].

Interleukin-10 is an immunoregulatory cytokine produced by type 2 T helper (Th2) cells and monocytes [159]. This cytokine has pleiotropic effects in immunoregulation, including downregulating expression of Th1 cytokines, enhancing B-cell proliferation and antibody production, and inhibiting the function of effector T-cells, macrophages and APCs [159]. In a report of six patients with urothelial carcinoma who received ipilimumab, a decrease in levels of IL-10 after treatment was documented in one patient who experienced an irAE of ischemic papillopathy and optic neuritis [89]. Similarly, reduced IL-10 production was reportedly associated with the antitumor response and irAEs in metastatic melanoma patients treated with anti-CTLA-4 ticilimumab [90].

Another cytokine associated with irAEs is interleukin-6 (IL-6), which plays a role in the acute-phase response of inflammation and in B-cell maturation [160]. Valpione and colleagues reported that a lower level of baseline circulating IL-6 was associated with increased irAE occurrence in melanoma patients treated with ipilimumab [53]. Additionally, a study of melanoma patients receiving ipilimumab treatment showed that lower levels of circulating IL-6, IL-8 and sCD25 at baseline were significantly correlated with a higher incidences of colitis [91]. In contrast, Tanaka et al. assessed the fluctuation of multiple cytokines in melanoma patients treated with nivolumab. The data indicated that increases in circulating IL-6 after treatment were significantly associated with the development of cutaneous irAEs [92]. Together, these results suggest that lower baseline IL-6 and an increase in IL-6 after ICI treatment may serve as predictive markers.

Chemokines which are chemotactic for activated T cells and involed in autoimmune and inflammatory diseases have been studied using multiplexed serum chemokine assays in order to identify potential biomarkers. Khan et al. evaluated 40 cytokines in 65 patients receiving immune checkpoint inhibitors. The results showed that significant increases in CXCL9, CXCL10, CXCL11 and CXCL13 occurred 2 weeks post treatment and in CXCL9, CXCL10, CXCL11, CXCL13, IL-10 and CCL26 at 6 weeks post treatment. Patients who developed irAEs had lower levels of CXCL9, CXCL10, CXCL11 and CXCL19 at baseline and exhibited greater increases in CXCL9 and CXCL10 levels posttreatment [93]. In addition, cytokine polymorphisms have been implicated in several autoimmune diseases [161]. However, the role of cytokine polymorphisms in susceptibility to irAEs is still poorly characterized.

CD163 is a scavenger receptor that is primarily expressed on monocytes and macrophages and can be shed into plasma as soluble CD163 (sCD163) by inflammatory stimuli [162]. Elevated levels of sCD163 have been found in a variety of autoimmune conditions, such as rheumatoid arthritis, pemphigus vulgaris, and multiple sclerosis [163–165]. A recent study revealed that an increased circulating sCD163 levels after nivolumab therapy was associated with the occurrence of irAEs [94].

Finally, the role of soluble CTLA-4 (sCTLA-4) in the identification of irAEs was also investigated in patients with metastatic melanoma (MM) receiving ipilimumab. The results showed that higher baseline sCTLA-4 levels were also associated with the onset of any irAE, in particular gastrointestinal irAEs [95].

### Blood Cells

Different subsets of peripheral blood cells are essential to host immunity. Changes in the blood cell population during immunotherapy have been extensively studied and found to be associated with the occurrence of irAEs. Fujisawa et al. found that increases in total white blood cell (WBC) count and decreases in relative lymphocyte count after treatment were associated with severe irAEs in univariate analyses [96]. Furthermore, the authors analyzed the correlation of changes in counts of WBCs and their subpopulations with organ-specific irAEs. The results showed that an increase in the total WBC count, a decrease in the relative lymphocyte count, and an increase in the relative neutrophil count were significantly correlated with the development of lung or gastrointestinal irAEs. A possible explanation is that neutrophilia may reflect an intensive response to systemic inflammation, whereas lymphopenia may suggest impaired cell-mediated immunity [166, 167]. In support of this finding, Shahabi et al. reported that elevated on-treatment expression of CD177 and carcinoembryonic antigen cell adhesion molecule 1 (CEACAM1), two neutrophil-activation markers, were correlated with GI irAEs, suggesting a possible role of neutrophils in ipilimumab-associated GI irAEs. In addition, expression of several immunoglobulin genes increased over time, with greater increases in patients with grade ≥ 2 GI irAEs [97].

Eosinophils are well known to contribute to the initiation and modulation of inflammation [168]. Growing evidence indicates an active role for eosinophils in several autoimmune disorders [168]. Infiltration of the thyroid gland with eosinophils is observed in patients with Hashimoto thyroiditis [169, 170]. In addition, increased serum concentrations of eosinophil-derived neurotoxins have been found in patients with Graves’ disease [171]. Therefore, eosinophils may also participate in the development of irAEs, and an increased eosinophil count may represent a biomarker. In a retrospective study of 167 patients with solid tumors, including melanoma, renal cell carcinoma, and urothelial carcinoma, treated with nivolumab or pembrolizumab, an absolute lymphocyte count > 2000 and elevated absolute eosinophil count at baseline were associated with the risk of irAE [98]. Likewise, another study of melanoma patients receiving anti-PD-1 antibodies showed that elevated absolute eosinophil count at baseline and relative eosinophil count at 1 month post treatment significantly correlate with the occurrence of endocrine irAEs, including ten cases of thyroid dysfunction, two cases of pituitary dysfunction, one case of primary adrenal insufficiency and one case of type 1 diabetes mellitus [99]. Increased eosinophil count has also been linked to irAEs.

Moreover, TCR repertoire, which can be broaden by immunotherapy, has been shown to correlate with the antitumor response and the development of irAEs [100, 172–174]. Oh and colleagues examined the correlation of irAEs with the repertoire of circulating T cells in patients with metastatic castration-resistant prostate cancer receiving a combination of ipilimumab and granulocyte-monocyte colony-stimulating factor [100]. Similar findings were reported by Subudhi et al. in a phase II clinical trial of androgen deprivation therapy plus ipilimumab in patients with castration-sensitive metastatic prostate cancer. TCR sequencing demonstrated that clonal expansion of CD8 T cell clones in peripheral blood preceded the onset of severe irAEs [101]. These results speak to the association of early T-cell diversification following initiation of ipilimumab with an increased risk of irAEs.

In summary, an elevated total white blood cell, absolute lymphocyte or eosinophil count at baseline, as well as the enlarged TCR repertoire, and a decrease in relative lymphocyte count after treatment, are related to an increased risk of irAEs.

### Gut Microbiome

The human gastrointestinal tract provides a niche for a complex microbial population, collectively referred to as the gut microbiota. The role of the gut microbiota in the pathogenesis of autoimmune diseases has earned substantial attention in recent years. Emerging studies suggest that the gut microbiota is implicated in the maintenance of immune homeostasis [175]. Certain microbial species, such as Bacteroides, Clostridium and Faecalibacterium, have been shown to induce expansion of T-regulatory cells or stimulate the production of anti-inflammatory cytokines [176–178]. These microbial species are essential for maintaining a tolerogenic state in the intestinal mucosa. Insights into the contribution of gut microbiota to the development of inflammatory bowel diseases (IBDs) have provided important information for the pathogenesis of colitis induced by ipilimumab. As with IBDs, dysbiosis also contributes to the development of colitis in patients receiving immunotherapy. Chaput et al. reported that baseline gut microbiota enriched with Faecalibacterium and other Firmicutes is associated with better clinical response to ipilimumab and higher risk of ipilimumab-induced colitis [91]. In contrast, administration of a combination of *B. fragilis* and *Burkholderia cepacia* induced protection against anti-CTLA4-induced intestinal lesions [179]. This protection was hypothesized to be due to proliferation of inducible T cell costimulator (ICOS) + Tregs in the lamina propria promoted by *B. fragilis*, possibly by mobilizing plasmacytoid DCs that accumulated and matured in mesenteric lymph nodes after *B. fragilis* monocolonization in germ-free mice receiving anti-CTLA4 Ab [43, 179, 180]. A prospective study of patients with metastatic melanoma undergoing ipilimumab treatment revealed that increased representation of bacteria belonging to the Bacteroidetes phylum at the start of treatment was associated with a reduced incidence of colitis [45]. Furthermore, the study sought to identify bacterial modules that can determine a patient’s risk for colitis. By analyzing the intestinal microbiota composition, it was found that a deficiency of four modules, which include a polyamine transport system module and modules involved in the biosynthesis of vitamins riboflavin (B2), pantothenate (B5) and thiamine (B1), predicts colitis risk with good accuracy. The recent report of successful treatment with fecal transplantation in two patients with ICI-induced colitis that was refractory to steroids, anti-TNF antibodies and anti-integrin antibodies also lends support to the role of intestinal microbiota in irAEs [181].

### Genetic Variability

Genetic predisposition is a contributing factor for susceptibility to autoimmunity. Distinct human leukocyte antigen (HLA) haplotypes and polymorphisms in immunoregulatory genes, such as CTLA4 and PD-1/PD-L1, are associated with a variety of autoimmune diseases [182–186]. Recently, the role of genetics or epigenetics, albeit less formally characterized, has been implicated in the development of irAEs. Stamatouli et al. reported a predominance of HLA-DR4 among patients treated with ICIs who developed autoimmune, insulin-dependent diabetes [102]. Bins et al. found that a homozygous variant of PDCD1 804 C > T (rs2227981) was associated with a decreased risk of irAEs in a study of 322 patients with NSCLC treated with nivolumab [103]. Despite these initial findings, future studies with larger patient cohorts will be required to identify the association between patient genetics and the development of irAEs.

## Future directions

Immunotherapy will form the backbone of therapy for various tumor types in the years to come. With more widespread clinical application of immune checkpoint inhibitors, knowledge of irAEs has continually evolved, and our understanding of the mechanisms of irAEs has provided deeper insight into the prediction and management of irAEs. However, several problems remain unsolved.

First, although several mechanisms have been proposed in the development of irAEs, the driver mechanisms, if any, that theoretically will play a central role in specific organ involvement are still largely unknown. It has been reported that the ectopic expression of CTLA-4 on the pituitary gland contributes to ICI-induced hypophysitis. However, in regard to irAEs of other organs, we can identify the potential driving inflammation pathway only based on the pathological analysis of the immune cell infiltration pattern of the involved organ, which may require a high-risk invasive procedure and time-consuming interpretation of the biopsy specimen. Therefore, the study of biomarkers could shed light on the underlying mechanisms and further potentially suggest therapeutic approaches, such as cytokine antagonists, to mitigate toxicities.

Second, as widely diverse irAE profiles are present in patients receiving immunotherapy, further studies should focus on how the interaction among immunotherapeutic agents, tumor histology types, and host genetic predisposition affects the development of irAEs. Despite the mechanisms of disrupted host T-cell tolerance induced by ICIs, the end-organ-specific adverse event pathophysiology needs further investigation. This will allow us to detect the onset of irAEs before clinical manifestations and to identify which subset of patients are likely to develop irAEs, what specific organs are prone to be involved, which is of great importance, particularly in patients with comorbid chronic diseases, such as cardiomyopathy or interstitial lung diseases.

Third, it is still premature to recommend the abovementioned biomarkers in the clinic due to the lack of prospective validation. To efficiently translate biomarkers into tests in clinical practice, researchers need to incorporate candidate biomarker testing into adequately powered and well-designed clinical trials. The overarching goal is to develop superior diagnostic tools that will facilitate the establishment of predictive models and early detection of irAEs. However, much is still unknown regarding the lack of uniform diagnostic criteria of irAEs and the extra layer of complexity added to the identification of irAEs when immunotherapy and chemotherapy or targeted therapy are used in combination.

## Conclusions

ICIs are frequently used and represent a new norm of care in some advanced cancers, which makes it important to better understand irAEs and their management. Biomarkers used to predict and track autoimmune toxicity in patients undergoing immunotherapy could serve to facilitate tailored monitoring, early identification and intervention, and customized therapy, even in patients with AID that are usually avoided. However, there are currently no validated biomarkers to select patients at the highest risk of developing irAEs or help to detect toxicities before presentation of clinical symptoms. Therefore, further larger and multi-institutional cohort studies are warranted to validate these potential biomarkers.

## Data Availability

Not applicable.
